# Britanin relieves ferroptosis-mediated myocardial ischaemia/reperfusion damage by upregulating GPX4 through activation of AMPK/GSK3β/Nrf2 signalling

**DOI:** 10.1080/13880209.2021.2007269

**Published:** 2021-12-03

**Authors:** Haoyang Lu, Hui Xiao, Manyu Dai, Yangcheng Xue, Ren Zhao

**Affiliations:** Department of Cardiovascular Medicine, the First Affiliated Hospital of Anhui Medical University, Hefei, China

**Keywords:** Myocardial infarction, hypoxia-reoxygenation injury, iron, apoptosis, oxidative stress

## Abstract

**Context:**

Ferroptosis was described as an important contributor to the myocardial ischaemia/reperfusion (MIR) injury, and britanin (Bri) was reported to exert antitumor and anti-inflammatory activities.

**Objective:**

Our study explores the effect and mechanism of Bri on MIR damage.

**Materials and methods:**

The rat model of MIR was established by ligation of the left anterior descending coronary artery. Male Sprague–Dawley (SD) rats were divided into three groups: sham group (*n* = 6), MIR group (*n* = 6) and MIR + Bri group (*n* = 6; 50 mg/kg). Rats were intragastrically pre-treated with Bri or normal saline once daily for 3 days. To further verify the role and mechanism of Bri, H9C2 cells were subjected to hypoxia plus reoxygenation (H/R) to induce the *in vitro* model of MIR.

**Results:**

Compared with MIR rats, Bri significantly decreased infarct area (22.50% vs. 38.67%), myocardial apoptosis (23.00% vs. 41.5%), creatine phosphokinase (0.57 U/mL vs. 0.76 U/mL), and lactate dehydrogenase levels (3.18 U/mL vs. 5.17 U/mL), concomitant with alleviation of ferroptosis. Mechanistically, Bri treatment induced the activation of the adenosine monophosphate activated protein kinase (AMPK)/glycogen synthase kinase 3β (GSK3β)/nuclear factor erythroid 2-related factor 2 (Nrf2) pathway *in vivo*. In addition, the AMPK/GSK3β/Nrf2 pathway participated in the regulation of glutathione peroxidase 4 (GPX4) expression, and silencing of Nrf2 attenuated the effect of Bri on H/R-induced cell injury.

**Discussion and conclusions:**

Bri protected against ferroptosis-mediated MIR damage by upregulating GPX4 through activation of the AMPK/GSK3β/Nrf2 signalling, suggesting that Bri might become a novel therapeutic agent for MIR.

## Introduction

Myocardial ischaemia and infarction caused by coronary artery disease is a major contributor to death throughout the world and has become a serious public health problem (Mortality and Causes of Death 2016). With myocardial infarction, reperfusion as soon as possible using thrombolytic therapy is the most effective method to relieve myocardial damage at present (Chi et al. 2017). But paradoxically, restoration of blood flow may induce inherent damage that causes myocardial death and increases the infarct size, which is termed as myocardial ischaemia/reperfusion (MIR) injury (Kalogeris et al. 2016). It follows that MIR insult is problematic in myocardial infraction therapy, and it is important to investigate ways to reduce MIR damage.

MIR damage was reportedly responsible for up to 50% of myocardial infarction. Hence, it is clinically significant to further explore MIR pathophysiology (Lillo-Moya et al. 2021). Extensive data have established that ferroptosis is a major driver of MIR injury (Wu et al. 2018, 2021). Ferroptosis is a type of cell death driven by iron-dependent lipid peroxidation (Stockwell et al. 2017). During the myocardial ischaemia period, the homeostasis of iron is disrupted, which causes the accumulation of free iron in mitochondria (Nakamura et al. 2019). The accumulation of metal ion triggers the Fenton reaction and Haber-Weiss reaction and then leads to the overgeneration of reactive oxygen species (ROS), resulting in lipid peroxidation and ferroptosis (Kobayashi et al. 2018). The occurrence of ferroptosis is accompanied by the decrease of glutathione (GSH) and glutathione peroxidase 4 (GPX4) levels. A previous study showed that during myocardial infarction, suppression of GPX4 by siRNA or RSL3 could cause the accumulation of lipid peroxide and result in ferroptosis in H9C2 cells, concluding that GPX4 protects against ferroptosis during myocardial infarction (Park et al. 2019). Ferroptosis was involved in doxorubicin-induced cardiotoxicity and MIR injury in mice, which was alleviated by inhibition of ferroptosis using ferrostatin-1 and iron chelation, highlighting that ferroptosis is a novel therapeutic target for MIR damage (Fang et al. 2019).

*Inula lineariifolia* Turcz. (Asteraceae)is ranked as a common medicinal plant in traditional Chinese medicine, which is widely used to modulate the function of the spleen, liver and stomach, and prevent abortion (Wang et al. 2014). Among the pharmacologically active phytochemicals, britanin (Bri) is a bioactive sesquiterpene lactone isolated from *Inula lineariifolia,* and has features of excellent anti-inflammatory, antioxidative and antitumor activities (Kim et al. 2016). In triple-negative breast cancer, Bri management could inhibit the proliferation, migration, and invasion of MDA-MB-231 and SUM-159 cells by inhibiting the nuclear factor-κB (NF-κB) pathway (Xu et al. 2020). Also, the antitumor activity of Bri in hepatocellular carcinoma progression has been documented. Bri repressed the colony formation ability and invasion of BEL 7402 and HepG2 cells, as well as reduced the tumour size in a BEL 7402-luc subcutaneous tumour model (Li, Du, et al. 2020). Additionally, Bri exerted its antitumor role in gastric cancer through NF-κB-mediated immune response (Shi et al. 2020). Besides, Bri was reported to protect against cerebral ischaemia-reperfusion damage by induction of the nuclear factor erythroid 2-related factor 2 (Nrf2) signalling pathway (Wu et al. 2017). However, there is still limited knowledge about the role of Bri in the pathological process of MIR damage.

This study elucidated the effect and molecular mechanisms of Bri on MIR damage, and found that Bri protected against MIR injury by inhibiting ferroptosis. Further mechanical experiments uncovered that Bri exerted its cardioprotective effects by upregulating GPX4 through activation of the adenosine monophosphate activated protein kinase (AMPK)/glycogen synthase kinase 3β (GSK3β)/Nrf2 signalling, providing a foundation for the clinical application of Bri.

## Materials and methods

### Animals

Male Sprague–Dawley rats (8-weeks-old, weighing from 250 to 280 g) were purchased from Shanghai Leigen Biotechnology Co., Ltd. (Shanghai, China), and handled in strict accordance with the Guide for Care and Use of Laboratory Animals published by the National Institute of Health. This study was approved by the Ethics Committee of the First Affiliated Hospital of Anhui Medical University.

### Myocardial ischemia–reperfusion (MIR) protocol

The rats were arbitrarily separated into three groups: sham group (*n* = 6), MIR group (*n* = 6) and MIR + Bri group (*n* = 6). The rats were given intraperitoneal anaesthesia with sodium pentobarbital (50 mg/kg).

During this surgery, we kept the rectal temperature at 37–38 °C employing a heating pad, and these rats received ventilation with a rodent ventilator. A PowerLab system with Chart 7.0 program was applied to record the electrocardiograph. We performed the thoracotomy to expose the heart, and then placed a tightened snare around the left anterior descending (LAD) coronary artery to occlude the artery. Myocardial ischaemia was established successfully when the ST segment of electrocardiogram was elevated and the colour of myocardial tissues was changed. After 30 min of occlusion, the snare was loosened to induce reperfusion for 24 h. At the end of the experiments, the blood samples were obtained from the rats. Then the rats were euthanized by decapitation, and the hearts were removed for the following experiments.

The rats in the sham group received the same procedures except for LAD occlusion. The rats in MIR + Bri group were intragastrically administered with Bri (50 mg/kg) once daily for 3 days, and the rat model of MIR was established 2 h after Bri administration on the third day.

### Infarcted area calculation

The myocardial tissues were excised, frozen, and sectioned into ∼1.5 mm slices. Then the slices were reacted for 15 min with 1% 2, 3,5 triphenyltetrazolium chloride (TTC) solution (Solarbio, Beijing, China), followed by incubation with 4% formaldehyde solution overnight. The slices were scanned, and the sizes of the ischaemic area (red) and infarct area (white) were analysed computationally.

### Determination of serum creatine phosphokinase (CPK) and lactate dehydrogenase (LDH) levels

After centrifugation, the serum was separated from the plasma. The cell culture medium was separated from H9C2 cells. The serum samples and cell culture medium were collected for the following experiments.

The levels of serum CPK were determined using a rat CPK ELISA kit (Xinyu, Shanghai, China) according to the manual of the product.

A LDH release assay kit from Beyotime (Shanghai, China) was utilized for the detection of LDH levels. The samples were incubated with LDH release reagent for 1 h and then the supernatant was collected after centrifugation. Following this, the samples were incubated with LDH detection solution for 30 min in dark, and the levels of LDH were determined by measuring the absorbance of each sample at 490 nm.

### Terminal-deoxynucleoitidyl transferase mediated nick end labeling (TUNEL) assay

The In Situ Cell Death Detection Kit (Roche, Penzberg, Germany) was applied to determine cell apoptosis. After fixation with 4% paraformaldehyde for 30 min, cell smears or tissue sections were washed with PBS solution, followed by permeation for 5 min with 0.3% Triton X-100. After washed with PBS, the samples were reacted with TUNEL reaction mixture for 1 h under the dark condition, followed by dyeing with 6-diamidino-2-phenylindole. Cell smears or tissue sections were pictured under a fluorescent microscope and the number of TUNEL-positive cells was analyzed computationally.

### Measurement of ROS, malondialdehyde (MDA), GSH and iron levels

The levels of ROS, MDA, GSH, and iron were measured using ROS assay kit, MDA assay kit, GSH assay kit, and iron assay kit according to the specifications.

#### Quantitative reverse transcription polymerase chain reaction (qRT-PCR)

RNA isolation was conducted using Trizol reagent (Life Technologies, CA, USA), and cDNA synthesis was done using iScript cDNA Synthesis kit (BioRad, CA, USA). The expression of GPX4 was measured with SYBR Green PCR Master Mix (Thermofisher Scientific, San Jose, CA, USA) and the following primers: GPX4 (forward: 5′-TGTGTAAATGGGGACGATGCC-3′; reverse: 5′-ACGCAGCCGTTCTTATCAATG-3′), and glyceraldehyde-3-phosphate dehydrogenase (GAPDH; forward: 5′-ACGGCAGGTTCAACGGCACAG-3′; reverse: 5′-CAGCATACTCAGCACCAGCATCAC-3′). The relative expression of GPX4 was normalized to that of GAPDH using 2^-ΔΔCt^ method.

### Western blot

The myocardial tissues were homogenized and then assayed for total protein extraction using a total protein extraction kit from Keygen (Nanjing, China). After sodium dodecyl sulphate polyacrylamide gel electrophoresis, the proteins were transferred to nitrocellulose membranes, and then immunoblotted with the primary antibodies, including GPX4 (1:1000), AMPK (1:1000), p-AMPK (1:1000), GSK3β (1:5000), p-GSK3β (1:5000), Nrf2 (1:2000), Lamin B (1:2000), and α-tubulin (1:5000). Following incubation with the peroxidase-conjugated secondary antibody, the blots were visualized employing an enhanced ECL chemiluminescence kit from Vazyme (Nanjing, Jiangsu, China), and analyzed using the Image J software (NIH Image, Bethesda, MD, USA). All the antibodies used in our study were from Abcam (Cambridge, MA, USA).

### Cell culture and treatment

H9C2 cells from the Cell Bank of the Chinese Academy of Sciences (Shanghai, China) were grown in Dulbecco’s modified Eagle medium (DMEM; Solarbio) containing 10% foetal bovine serum (FBS; Solarbio) and 1% penicillin/streptomycin at 37 °C in an atmosphere containing 5% CO_2_ in the air. The medium was changed every 2 days, and the cells were cultured until 80%∼90% confluent.

Following digestion with 0.05% trypsin, H9C2 cells were planted into cell culture plates and transfected with si-GPX4, si-AMPK, si-Nrf2, GSK3β-OE or the corresponding controls. After 12 h of transfection, the cells were pre-treated with Bri (5 μM) for 6 h.

To mimic MIR culture condition, H9C2 cells were cultured in glucose-free and serum-free DMEM medium in an atmosphere containing 1% O_2_, 5% CO_2_, and 94% N_2_ at 37 °C. At 3 h after hypoxic exposure, H9C2 cells were incubated for 24 h in DMEM medium plus 10% FBS at 37 °C in a humidified incubator (5% CO_2_/95% air) to reoxygenate.

### Cell count kit-8 (CCK-8) assay

After transfection, H9C2 cells were planted into 96-well plates and cultured at 37 °C in an atmosphere containing 5% CO_2_ in the air. After 24 h of incubation, H9C2 cells were treated with Bri (5 µM) for 6 h, followed by exposure to H/R condition. Following this, CCK-8 solution from Vazyme was added into each well and cultured for 2 h. The absorbance of each sample at 450 nm was detected to assess cell viability.

### Statistical analysis

Our data were given as mean ± standard error and analysed using one-way analysis of variance followed by Turkey’s multiple-comparison test with Prism v8 (GraphPad, La Jolla, CA, USA). The difference was deemed significant when a *p*-value of <0.05 was obtained.

## Results

### Bri protected against MIR injury

**Figure 1.**
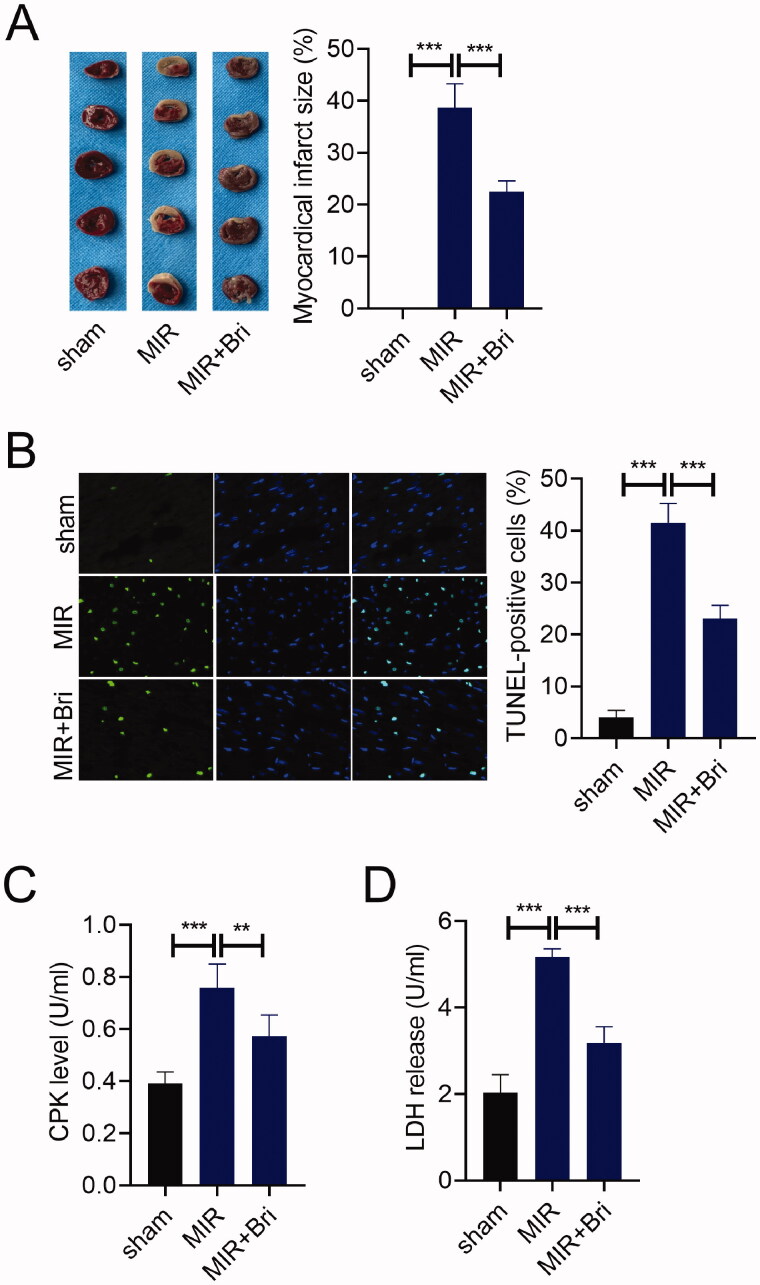
Bri protected against MIR injury. SD rats were intragastrically administered with Bri, followed by undergoing MIR surgery. (A) Representative images and quantification of myocadiac infarction area in the sham, MIR, and MIR + Bri group. (B) Representative images and quantification of myocardial apoptosis in the sham, MIR, and MIR + Bri group. (C and D) Commercial kits were used to analyse the levels of serum CPK and LDH in the sham, MIR, and MIR + Bri group. **p < 0.01 and ***p < 0.001.

First, we study the impact of Bri on MIR injury *in vivo*. The infarct size of the heart from MIR rats was determined by TTC staining, and we found that no myocadiac infarction was noticed in the sham group, and a serious myocadiac infarction was found in the MIR group, which was relieved by Bri treatment (Figure 1(A)). The number of TUNEL-positive cells in the MIR group was much higher than that in the sham group. Bri treatment could reduce the number of TUNEL-positive cells in myocardium of MIR rats (Figure 1(B)). Moreover, the levels of serum CPK were increased in the MIR group compared with that in the sham group, but were reduced in the MIR + Bri group compared with that in the MIR group (Figure 1(C)). Consistently, compared with the sham group, the levels of serum LDH were significantly elevated in the MIR group. Bri management could attenuate MIR-induced elevation of LDH levels (Figure 1(D)).

### Bri alleviated MIR-induced ferroptosis

**Figure 2.**
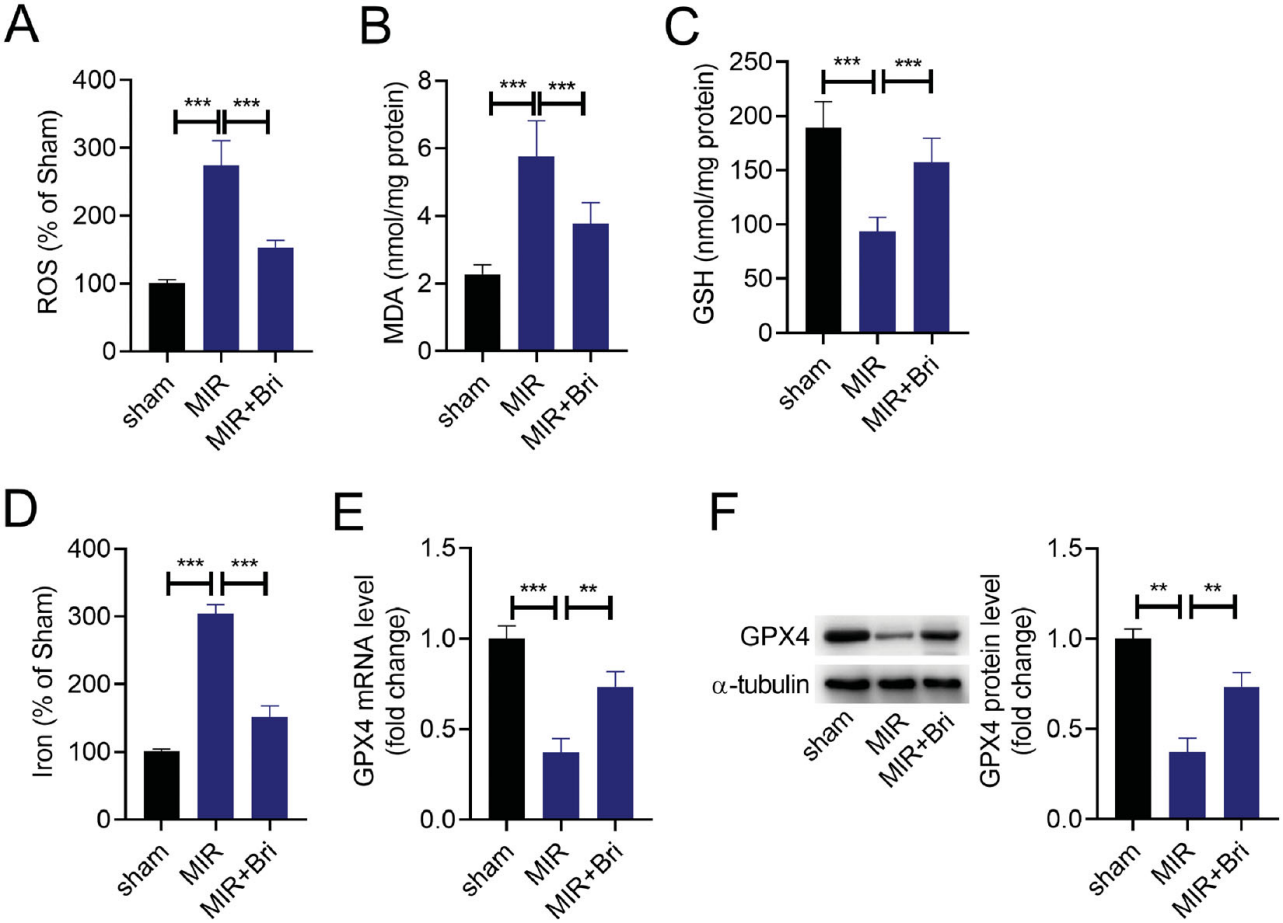
Bri alleviated MIR-induced ferroptosis. SD rats were intragastrically administered with Bri, followed by undergoing MIR surgery. The levels of ROS (A), MDA (B), GSH (C), and iron (D) in the myocardium of rats were determined using commercial kits. (E and F) qRT-PCR and western blot analysis of GPX4 expression in the myocardium of rats. **p < 0.01 and ***p < 0.001.

Next, we explored whether Bri protected against MIR injury by regulating ferroptosis. First, we detected the levels of ROS, MDA, GSH, and iron in the myocardium of rats by corresponding commercial kits. As shown in Figure 2(A–D), the levels of ROS, MDA, and iron were increased in the myocardium of MIR rats compared with those in the myocardium of sham rats, and these increases were mitigated after Bri administration. Conversely, the level of GSH was strikingly decreased in the myocardium of MIR rats, which was attenuated following Bri treatment. In line with this, a reduction of GPX expression level was noticed in the myocardium of MIR rats, as determined by qRT-PCR and western blot. These results suggested that Bri management increased the levels of GPX4 in MIR rats (Figure 2(E,F)).

### Bri inhibited ferroptosis-dependent H/R injury by facilitating GPX4 expression *in vitro*

**Figure 3.**
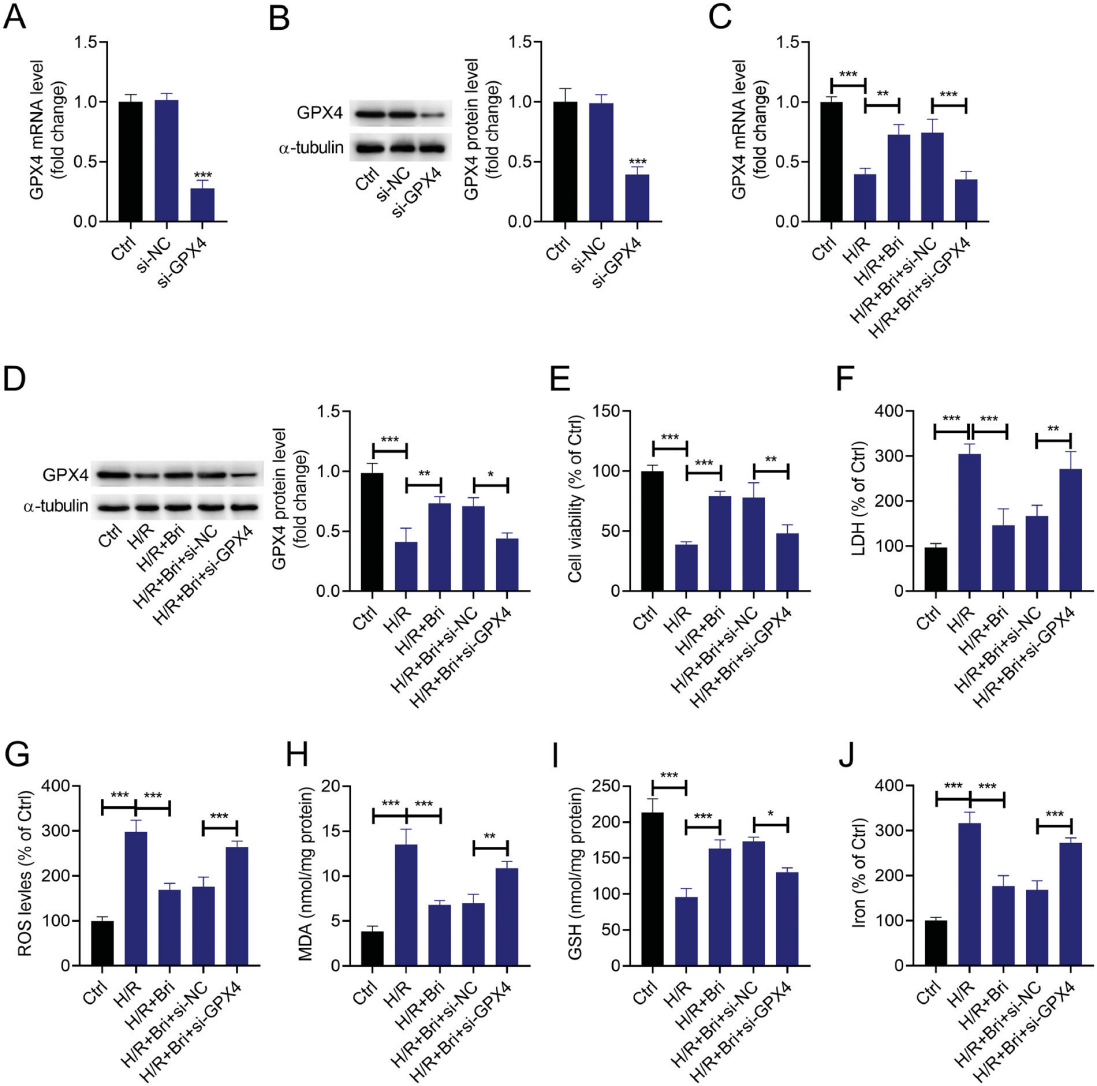
Bri inhibited ferroptosis-dependent H/R injury by facilitating GPX4 expression *in vitro*. H9C2 cells were transfected with si-GPX4 or si-NC and then treated with Bri. After 6 h of treatment, H9C2 cells were exposed to H/R condition. (A and B) qRT-PCR and western blot analysis of GPX4 expression in H9C2 cells transfected with si-GPX4 or si-NC. (C and D) GPX4 expression was detected in H9C2 cells with different treament. (E) The viability of H9C2 cells was determined using CCK-8 assay. (F) LDH release assay was conducted to determine the level of LDH in H9C2 cells. The levels of ROS (G), MDA (H), GSH (I), and iron (J) in H9C2 cells were determined using commercial kits. *p < 0.05, **p < 0.01, and ***p < 0.001.

To affirm the cardioprotective effect of Bri on MIR damage *in vitro*, H9C2 cells were transfected with si-GPX4 or si-NC and then treated with Bri, followed by exposure to H/R condition. As determined by qRT-PCR and western blot, a marked reduction of GPX4 expression level was observed in H9C2 cells transfected with si-GPX4 compared with that in H9C2 cells transfected with si-NC (Figure 3(A,B)). Compared with the H/R group, Bri treatment increased the expression levels of GPX4 mRNA and protein in H9C2 cells, and this effect of Bri was reversed by transfection of si-GPX4 (Figure 3(C,D)). H/R exposure inhibited the viability of H9C2 cells, and this inhibition was weakened in the presence of Bri. Knockdown of GPX4 could abrogate Bri-induced increase of cell viability in H9C2 cells under H/R condition (Figure 3(E)). Similarly, Bri administration restrained H/R-induced elevation of LDH levels in H9C2 cells, and this effect could be counteracted after downregulation of GPX4 (Figure 3(F)). Besides, Bri administration restrained H/R-induced increase in ROS, MDA, and iron levels and decrease in GSH level in H9C2 cells. These changes induced by Bri treatment were impaired after silencing of GPX4 (Figure 3(G–J)).

### Bri exerted regulatory effects on the AMPK/GSK3β/Nrf2 pathway in MIR rats

**Figure 4.**
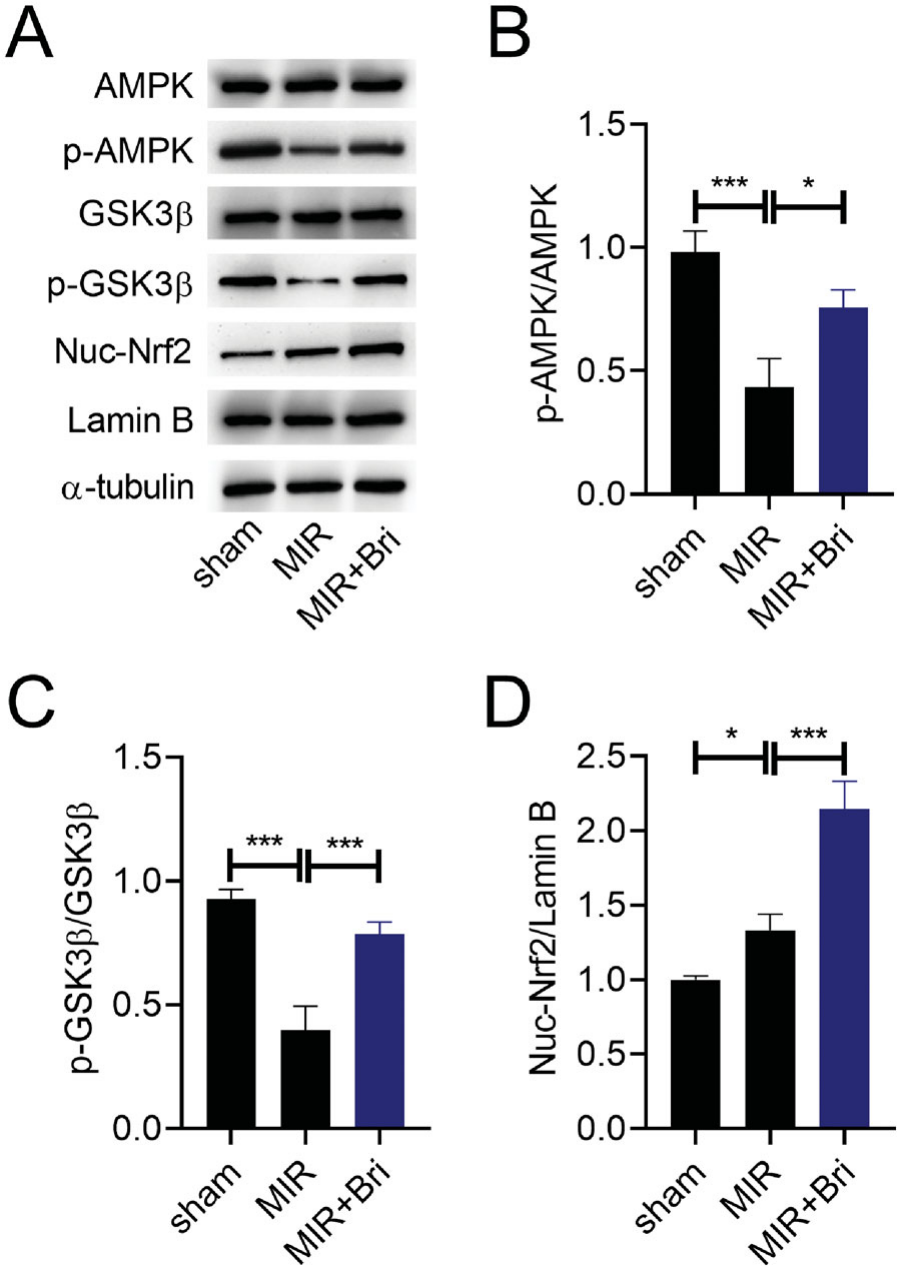
Bri exerted regulatory effects on the AMPK/GSK3β/Nrf2 pathway in MIR rats. SD rats were intragastrically administered with Bri, followed by undergoing MIR surgery. (A) Representative images and (B–D) quantification of western blot results of AMPK, p-AMPK, GSK3β, p-GSK3β, and Nuc-Nrf2 expression in the myocardium of MIR rats. *p < 0.05 and ***p < 0.001.

To study how Bri exerts its cardioprotective effect in MIR rats, we evaluated the expression of AMPK, p-AMPK, GSK3β, p-GSK3β, nuclear (Nuc)-Nrf2, and Lamin B in the myocardium of MIR rats using western blot. As a result, the expression levels of p-AMPK and p-GSK3β were reduced, but the expression level of Nuc-Nrf2 was increased in the myocardium of MIR rats. Bri treatment increased the expression levels of p-AMPK and p-GSK3β, but decreased the expression level of Nuc-Nrf2 in the myocardium of MIR rats (Figure 4(A)). Consistently, the ratios of p-AMPK/AMPK and GSK3β/p-GSK3β were reduced in the myocardium of MIR rats, but these reductions were weakened after Bri administration (Figure 4(B,C)). Conversely, the ratio of Nuc-Nrf2/Lamin B was increased in the myocardium of MIR rats, which was enhanced by Bri administration (Figure 4(D)).

### The AMPK/GSK3β/Nrf2 pathway was involved in the regulation of GPX4 expression

**Figure 5.**
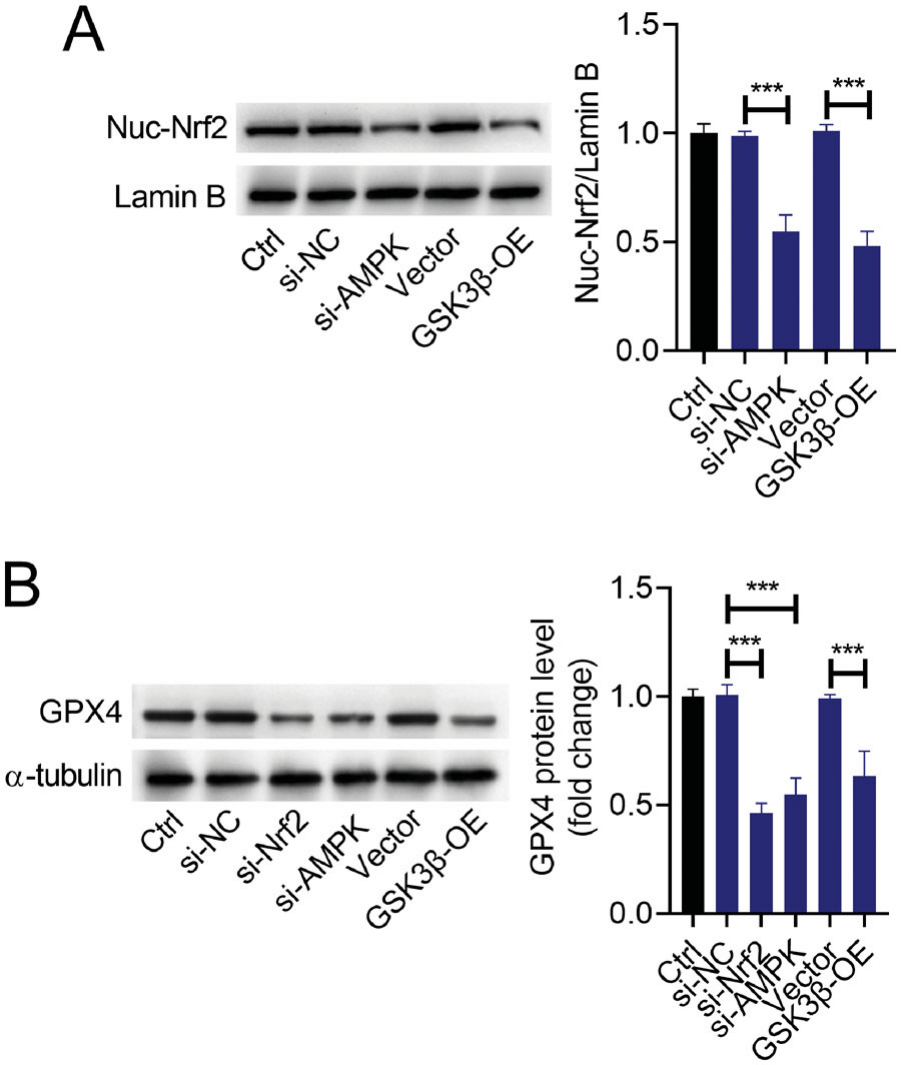
The AMPK/GSK3β/Nrf2 pathway was involved in the regulation of GPX4 expression. H9C2 cells were transfected with si-AMPK, si-Nrf2, GSK3β-OE or matched controls. Western blot analysis of Nuc-Nrf2 (A) and GPX4 (B) expression in H9C2 cells. ***p < 0.001.

To verify if the AMPK/GSK3β/Nrf2 pathway participates in the regulation of GPX4, we transfected si-AMPK, si-Nrf2, GSK3β-OE, or matched controls into H9C2 cells. The ratio of Nuc-Nrf2/Lamin B was reduced after si-AMPK or si-Nrf2 transfection. Similarly, upregulation of GSK3β reduced the ratio of Nuc-Nrf2/Lamin B in H9C2 cells (Figure 5(A)). Furthermore, either silencing of AMPK or Nrf2 reduced the expression level of GPX4 in H9C2 cells. Also, upregulation of GSK3β showed a similar effect on GPX4 expression in H9C2 cells (Figure 5(B)).

### Nrf2 attenuated the effect of Bri on H/R-induced cell injury in H9C2 cells

**Figure 6.**
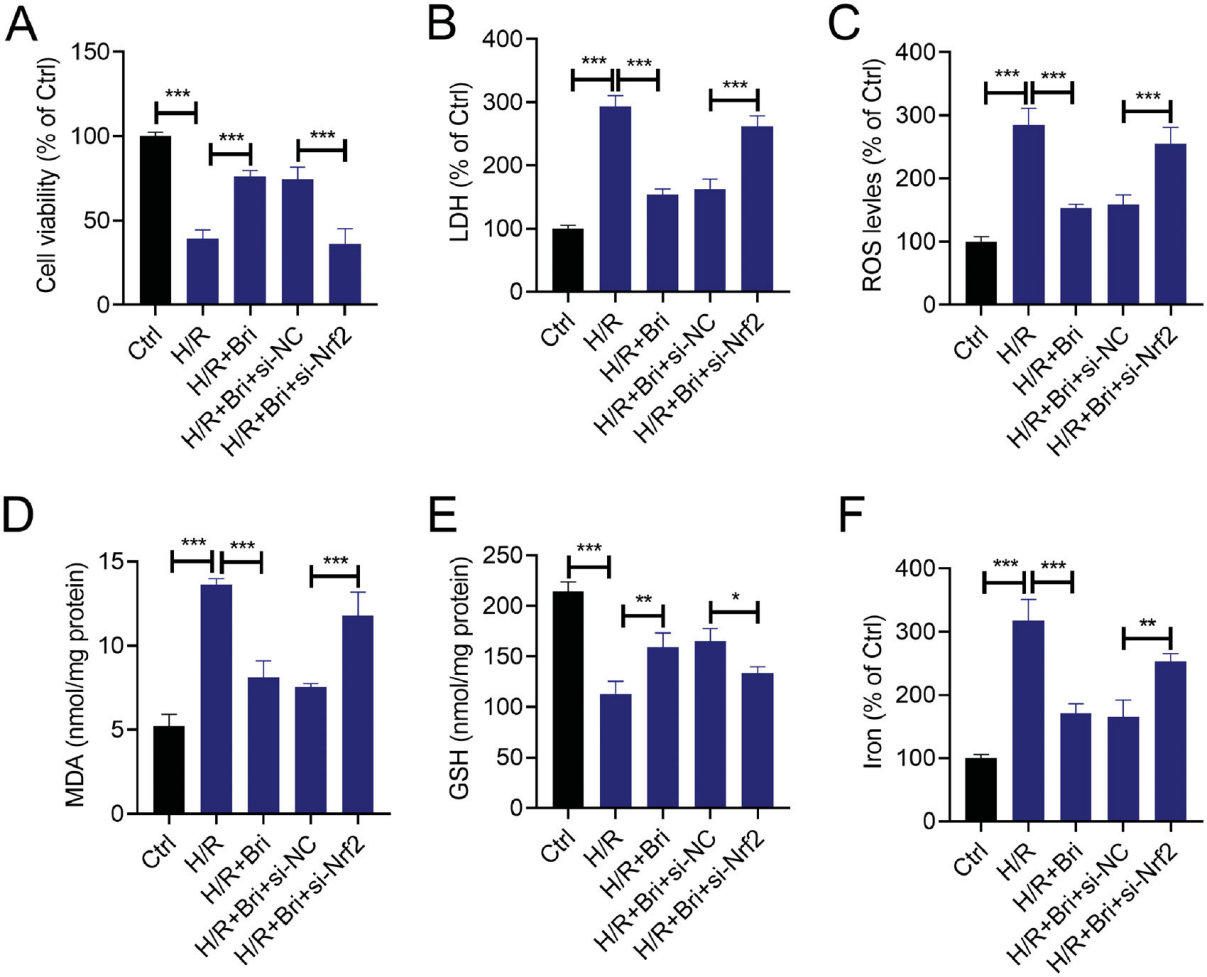
Nrf2 attenuated the effect of Bri on H/R-induced cell injury in H9C2 cells. H9C2 cells were transfected with si-Nrf2 or si-NC. At 18 h after transfection, H9C2 cells were treated with Bri, followed by induction of H/R injury. (A) The viability of H9C2 cells was determined using CCK-8 assay. (B) LDH release assay was conducted to determine the level of LDH in H9C2 cells. The levels of ROS (C), MDA (D), GSH (E), and iron (F) in H9C2 cells were determined using commercial kits. *p < 0.05, **p < 0.01, and ***p < 0.001.

To further investigate whether the cardioprotective effect of Bri on H/R-induced cell injury was mediated by Nrf2, we transfected si-Nrf2 or si-NC into H9C2 cells. At 18 h after transfection, H9C2 cells were treated with Bri, followed by subjected to H/R condition. As indicated by the result of CCK-8 assay, silencing of Nrf2 attenuated Bri-caused the increase of cell viability in H/R-stimulated H9C2 cells (Figure 6(A)). In H/R-stimulated H9C2 cells, Bri-induced the reduction of LDH level was counteracted after downregulation of Nrf2 (Figure 6(B)). Besides, Bri administration restrained H/R-induced increases in ROS, MDA, and iron levels and decrease in GSH level in H9C2 cells, while, these changes induced by Bri treatment were weakened after silencing of Nrf2 (Figure 6(C–F)).

## Discussion

MIR damage was described as a principal pathomechanism of myocardial infarction, therefore, there is an urgent need to find an agent that prevents MIR injury. Bri is a novel discovered compound that is investigated by researchers because of its biological activities. As a tumour-suppressing drug, Bri was proven to restrain the growth of human prostate cancer cell line PC-3-LUC by regulating phosphatidylinositol 3-kinase/Akt/NF-κB signalling and activating antitumor immunity by upregulating the level of interleukin-2 (Zeng et al. 2020). Also, evidence from both *in vivo* and *in vitro* experiments suggested that Bri hindered the proliferation and migration of pancreatic cancer cells by inducing the inactivation of NF-κB (Li, Zhou, et al. 2020). Apart from its antitumor activity, Bri was found to protect against cerebral ischaemic reperfusion damage by activating the Nrf2 signalling pathway (Wu et al. 2017). Nevertheless, there is no research available regarding the role of Bri in preventing MIR damage. Herein, we found that Bri management protected against MIR damage, and alleviated MIR-induced ferroptosis. Moreover, Bri inhibited ferroptosis-dependent H/R injury by facilitating GPX4 expression *in vitro*, suggesting the cardioprotective effect of Bri on MIR injury.

AMPK is a key regulator of lipid metabolism and glucose metabolism, and plays a prominent part in the regulation of cellular energy homeostasis (Li et al. 2017). It was reported that the impairment of AMPK phosphorylation appeared to influence the uptake and metabolism of glucose in the heart during myocardial ischaemia, revealing that AMPK might be a strategic target for myocardial infarction (Gu et al. 2018). GSK3β is an ubiquitously expressed serine/threonine kinase that plays several momentous roles in the heart. Phosphorylation of GSK3β could relieve MIR damage, concluding that inhibition of GSK3β is a cardioprotective mechanism of MIR injury (Ghaderi et al. 2017). Nrf2 is recognized as a vital factor in response to oxidative stress (Vomund et al. 2017). Under the pathophysiological situation, activated-Nrf2 that dissociated from Keap1, translocates into cell nucleus and then binds to antioxidant response element (ARE), which regulates the expression of ARE-mediated antioxidant enzyme genes (Barančik et al. 2016; Ding et al. 2020; Fei et al. 2020). Emerging evidence suggests that AMPK can promote the nuclear export of Nrf2 by inducing the phosphorylation of GSK3β (Lv et al. 2017). A previous study suggested that the AMPK/GSK3β/Nrf2 signalling taken part in the protective effect of butin against MIR injury in diabetic mice. Inhibition of the AMPK/GSK3β/Nrf2 signalling by Nrf2 silencing could impair the protective effect of butin on MIR injury, implying the involvement of the AMPK/GSK3β/Nrf2 signalling in MIR injury (Duan et al. 2017). However, whether the AMPK/GSK3β/Nrf2 signalling participates in the cardioprotective effect of Bri on MIR damage has never been elucidated. In this study, Bri treatment promoted the phosphorylation of AMPK and GSK3β and increased the level of nuc-Nrf2 in myocardium of MIR rats, revealing the involvement of the AMPK/GSK3β/Nrf2 signalling in the protective effect of Bri on MIR injury.

As AMPK/GSK3β/Nrf2 signalling was found to mediate the cardioprotective effect of Bri on MIR damage, we further determined how the AMPK/GSK3β/Nrf2 signalling participated in the protective effect of Bri on MIR injury. Previous studies have demonstrated that the AMPK/GSK3β/Nrf2 signalling appeared to function as a key regulator of ferroptosis. AMPK was reported as a key player in the regulation of ferroptosis: inactivation of AMPK could aggravate renal ischaemia-reperfusion injury *in vivo* by promoting ferroptosis (Lee et al. 2020). In addition, upregulation of GSK-3β augmented erastin-induced ferroptosis in breast cancer by upregulating the expression of GPX4, and this augment was blocked by activation of Nrf2, indicating that the GSK-3β/Nrf2 signalling controls ferroptosis by regulating the expression of GPX4 (Wu et al. 2020). Based on this evidence, we inferred that Bri attenuated ferroptosis-mediated MIR injury by upregulating GPX4 via the AMPK/GSK3β/Nrf2 signalling. In the present study, we found that upregulation of GSK3β reduced the level of Nuc-Nrf2 in H9C2 cells. Moreover, both silencing of AMPK or Nrf2 and upregulation of GSK3β reduced the expression level of GPX4 in H9C2 cells, indicating that the AMPK/GSK3β/Nrf2 signalling participated in the protective effect of Bri on MIR injury by upregulating the expression of GPX4.

As a nuclear transcription factor, Nrf2 is capable of regulating the expression of lipid peroxidation-associated genes, thereby controlling ferroptosis (Dodson et al. 2019). As an example, activation of Nrf2 reportedly interacted transcriptional coactivator small v-maf avian musculoaponeurotic fibrosarcoma oncogene homolog proteins to impair erastin- and sorafenib-induced ferroptosis in hepatocellular carcinoma cells (Sun et al. 2016). Furthermore, Nrf2 relieved acute lung injury and intestinal ischaemia reperfusion damage by inhibiting ferroptosis, suggesting that Nrf2 acted as a central switch in regulating ferroptosis (Dong et al. 2020). In this research, we found that silencing of Nrf2 attenuated the effect of Bri on H/R-induced cell injury in H9C2 cells, revealing that Bri protected against ferroptosis-mediated MIR damage through inducing the activation of AMPK/GSK3β/Nrf2 signalling.

## Conclusions

Taken all together, we presented evidence that Bri exerted a cardioprotective effect on MIR insult. Mechanically, Bri treatment upregulated the expression of GPX4 through AMPK/GSK3β/Nrf2 signalling, and as a result, attenuated ferroptosis-mediated MIR damage. Our findings revealed that Bri might be developed as a drug for clinical treatment of MIR damage.
